# Supplementary material for: Anti-miRNA103/107 encapsulated in transferrin-conjugated lipid nanoparticles crosses blood-brain barrier and reduces brain ischemic damage
Source: Mol Ther Nucleic Acids. 2024 Feb 2;35(1):102131. doi: 10.1016/j.omtn.2024.102131 (PMC10877170; doi:10.1016/j.omtn.2024.102131)
Supplement: Document S1. Figures S1–S3 and Table S1 [file mmc1.pdf]

OMTN, Volume *35*

Supplemental information

Anti-miRNA103/107 encapsulated in transferrin-conjugated lipid nanoparticles crosses blood-brain barrier and reduces brain ischemic damage

Pasquale Cepparulo, Ornella Cuomo, Virginia Campani, Antonio Vinciguerra, Maria Josè Sisalli, Valeria Nele, Serenella Anzilotti, Valeria Valsecchi, Antonella Casamassa, Paola Brancaccio, Antonella Scorziello, Giuseppe De Rosa, Lucio Annunziato, and Giuseppe Pignataro

Figure S1

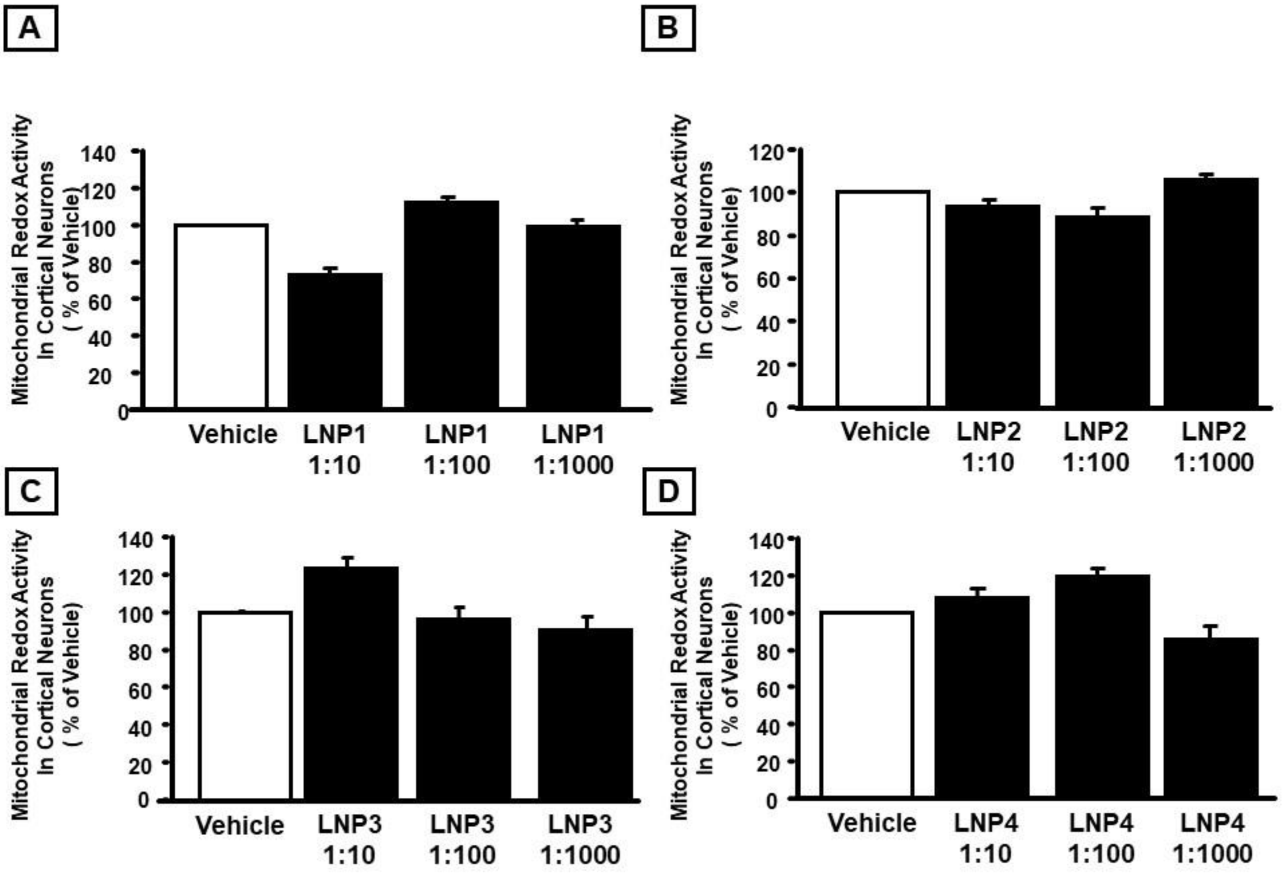

Figure S1. Effect of the different LNPs formulations at 1/100, 1/100 and 1/10 dilution filled with miRNA scramble on Mitochondrial Redox Activity in Cortical Neurons.

Figure S2

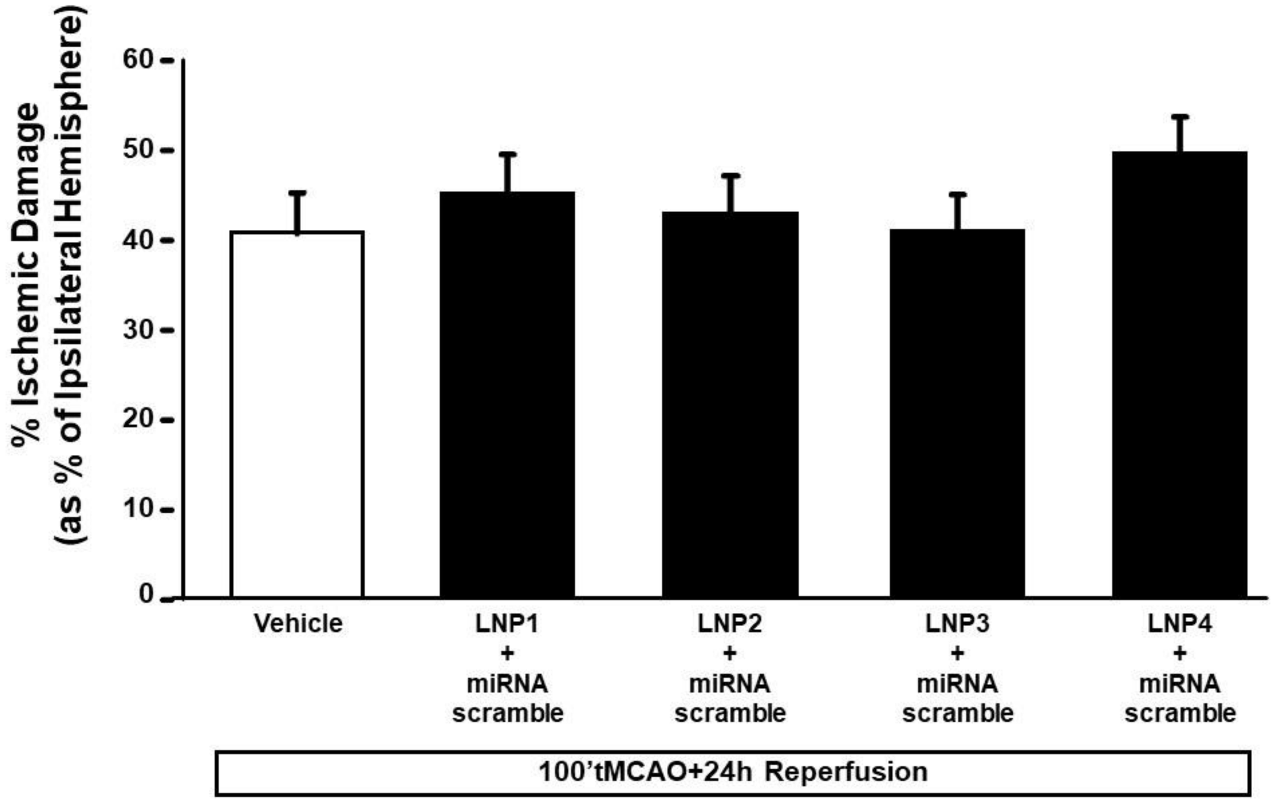

Figure S2. In vivo effect of LNP1-4 filled with miRNA scramble on ischemic volume of rats subjected to tMCAO followed by 24 hours from reperfusion.

Ischemic damage was assessed in rats subjected to tMCAO and treated with four in bolus injections of LNPs through the caudal vein 24 and 18 hours before tMCAO induction, and 1 and 5 hours after reperfusion. Vehicle group represents animals treated with tMCAO and saline solution. Each column represents the mean ± S.E.M.

Figure S3

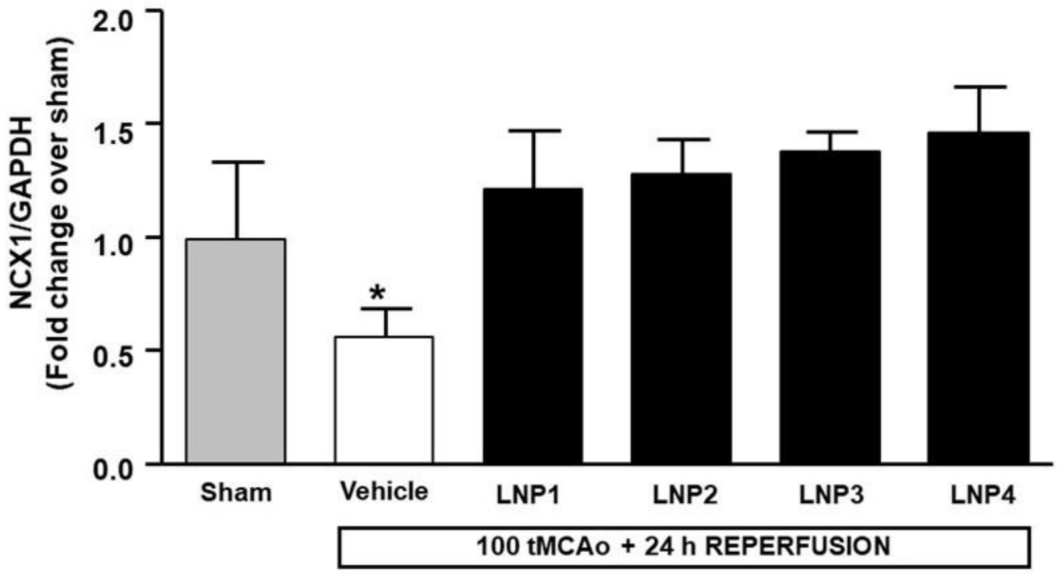

Figure S3. Evaluation of NCX1 expression in samples of ipsilateral striatum from ischemic rats treated with anti-miRNA103/107-loaded LNPs.

ncx1 mRNA levels are expressed as fold change of relative expression levels over the sham-operated group. Each column represents the mean ± S.E.M. Results of ncx1 expression were normalized with respect to GAPDH as internal control. n=3 samples per each group.

*: $p<0.05$ Vs control group.

Table S1: Original data corresponding to data presented in Figures 2, 4 and 5

Fig.2A

Vehicle	0.9±0.2
LNP1	0.57±0.06
LNP2	0.91±0.2
LNP3	0.93±0.23
LNP4	0.87±0.071

Fig.2B

Vehicle	4.4±1.2
LNP1	2128±193
LNP2	2702±279
LNP3	1472±582
LNP4	2732±394

Fig.4A antimiR expression in cortex

Vehicle	0.77±0.2
LNP1	20.9±10.5
LNP2	13.2±7.2
LNP3	10.9±4.7
LNP4	2.6±1.1

Fig.4C NCX1 mRNA expression in cortex

Sham	1.04±0.03
Vehicle+tMCAO	0.47±0.01
LNP1+tMCAO	0.71±0.09
LNP2+tMCAO	0.48±0.16
LNP3+tMCAO	1.01±0.27
LNP4+tMCAO	0.48±0.16

Fig5A AntimiR expression in plasma

0	1.93±0.9
3h	24875±4241
24h	2420±11.91
48h	322±168
96h	388±58
168h	0.6±0.24
