# Supplementary material for: Anti-miRNA103/107 encapsulated in transferrin-conjugated lipid nanoparticles crosses blood-brain barrier and reduces brain ischemic damage
Source: Mol Ther Nucleic Acids. 2024 Feb 2;35(1):102131. doi: 10.1016/j.omtn.2024.102131 (PMC10877170; doi:10.1016/j.omtn.2024.102131)
Supplement: Document S2. Article plus supplemental information [file mmc2.pdf]

Molecular Therapy
Nucleic Acids
Original Article

Anti-miRNA103/107 encapsulated in transferrin-conjugated lipid nanoparticles crosses blood-brain barrier and reduces brain ischemic damage

Pasquale Cepparulo,[1,6] Ornella Cuomo,[1,6] Virginia Campani,[2,6] Antonio Vinciguerra,[3] Maria Josè Sisalli,[1] Valeria Nele,[4] Serenella Anzilotti,[4] Valeria Valsecchi,[1] Antonella Casamassa,[5] Paola Brancaccio,[1] Antonella Scorziello,[1] Giuseppe De Rosa,[2] Lucio Annunziato,[5] and Giuseppe Pignataro[1]

[1]Division of Pharmacology, Department of Neuroscience, School of Medicine, "Federico II" University of Naples, Via Pansini, 5 - 80131 Naples, Italy; [2]Department of Pharmacy, University Federico II of Naples, Via Domenico Montesano, 49 - 80131 Naples, Italy; [3]Department of Biomedical Sciences and Public Health, School of Medicine, University "Politecnica delle Marche", 60126 Ancona, Italy; [4]Department of Science and Technology, University of Sannio, 82100 Benevento, Italy; [5]SYNLAB SDN, Via Gianturco, 113 - 80143, Napoli, Italy

MicroRNA (miRNA), by post-transcriptionally regulating the expression of genes involved in stroke response, represents important effectors in stroke pathophysiology. Recently, the 103/107 miRNA family emerged as a possible therapeutic target in stroke, as it controls the expression of sodium calcium exchanger 1, a plasma membrane transporter that plays a fundamental role in stroke pathophysiology. Although the neuroprotective properties of this and other miRNAs are promising, several pharmacokinetic drawbacks remain to be faced for the development of a translatable therapy based on small RNAs in CNS diseases. In the present study, to overcome these limitations, the anti-miRNA103/107 was encapsulated in specific preparations of lipid nanoparticles (LNPs), and their effectiveness was evaluated both in an *in vitro* model of hypoxia represented by primary neuronal cortical cultures exposed to oxygen and glucose deprivation followed by reoxygenation, and in an *in vivo* model of stroke obtained in rats exposed to transient occlusion of the middle cerebral artery. The results of the present study demonstrated that the encapsulation of anti-miRNA103/107 in transferrin-conjugated PEG-stabilized LNPs allowed the blood-brain barrier crossing and significantly reduced brain ischemic damage. The present achievements pave the way for the exploitation of a systemic intravenous miRNA delivery strategy in stroke therapy.

INTRODUCTION

Despite the advancement of knowledge in the field of therapy for neurodegenerative diseases and cerebral ischemia, the results so far obtained are not completely satisfactory. Indeed, despite that cerebral ischemia represents the second cause of death and the leading cause of disability in the world, the tissue plasminogen activator (rTPA) remains the only therapeutic option, and its use is limited to 3%–4% of patients, for its very narrow therapeutic time window.[1] Considering these premises, it urges the need to develop innovative therapeutic strategies capable of overcoming the limits of the available treatment. In this regard, a class of small non-coding RNA, microRNA or miRNA molecules, are emerging as promising therapeutic options in stroke and other neurological disorders.[2] In fact, these molecules can regulate the expression of target proteins involved in the pathogenesis of the entire spectrum of known disorders including neoplastic, cardiovascular, infectious, degenerative, and inflammatory-autoimmune diseases.[3] What make miRNAs particularly attractive as potential therapeutic agents is the ability to target at the same time more than 20 different messenger RNAs, either preventing their translation, or promoting their degradation, thus regulating the activity of numerous proteins.[4,5] This latter aspect is particularly relevant for pathologies characterized by the activation of several pathways that cannot be targeted by a single drug.

Recently, the 103/107 miRNA family has been identified as a possible therapeutic target in stroke, as it is able to determine the downregulation of the member 1 of sodium calcium plasma membrane exchanger family (NCX1), which plays a fundamental role in stroke.[6–11] The use of the specific anti-miRNA, able to prevent stroke-induced NCX1 downregulation determined a significant reduction of the infarct lesion in ischemic rats.[12] Although the neuroprotective properties of this and other miRNAs are promising, several pharmacokinetic drawbacks remain to be faced for the development of a translatable therapy based on small RNAs in CNS diseases, such

Received 9 February 2023; accepted 29 January 2024;
https://doi.org/10.1016/j.omtn.2024.102131.

[6]These authors contributed equally

Correspondence: Giuseppe Pignataro, MD, PhD, Division of Pharmacology, Department of Neuroscience, School of Medicine, "Federico II" University of Naples, Via Pansini 5, 80131 Naples, Italy.
E-mail: giuseppe.pignataro@unina.it

This is an open access article under the CC BY license (http://creativecommons.org/licenses/by/4.0/).

Table 1. LNP-anti-miR103/107composition

Lipid composition of LNPs (molar ratio)							
Formulation	DSPC	CHOL	DODAP	PEG2000-Cer16	DSPE-PEG-Mal	Surface conjugation	Anti-miR103/107theoretical loading (nmol/mg lipids)
LNP	1	1.8	0.8	0.4	–	–	–
LNP 1	1	1.8	0.8	0.4	–	–	27.6
LNP 2	1	1.8	2	0.4	–	–	41.4
LNP 3	1	1.8	0.8	0.2	0.2	Transferrin	27.6
LNP 4	1	1.8	2	0.2	0.2	Transferrin	41.4

as (1) stability after systemic administration, (2) blood-brain barrier (BBB) crossing, (3) achievement of the ischemic brain region, and (4) uptake into the target cells.

To overcome these limitations, the anti-miRNA103/107 was encapsulated in specific preparations of lipid nanoparticles (LNPs), including transferrin-conjugated stabilized lipid nanoparticles, and their effectiveness was evaluated both in an *in vitro* model of hypoxia represented by primary neuronal cortical cultures exposed to oxygen and glucose deprivation (OGD) followed by reoxygenation, and in an *in vivo* model of stroke obtained in rats exposed to transient occlusion of the middle cerebral artery (tMCAO).

The development of this delivery approach of 103/107 anti-miRNA capable of specifically reaching the CNS following systemic administration may therefore constitute an innovative therapeutic strategy for the treatment of ischemic brain disease both in terms of drug molecular structure and in terms of pharmaceutical formulation.

RESULTS

Characterization of LNPs encapsulating anti-miR-103/107

Different lipid nanoparticle (LNP) formulations were prepared (Table 1) and characterized as described in the materials and methods section. As reported in Table 2, LNPs prepared without anti-miR103/107 had a mean diameter of about 147.7 nm and a negative zeta potential (ζ) (−16.8 mV). The encapsulation of anti-miR103/107 as well as the conjugation of transferrin (Tf) on the LNP surface led to an increase of the size up to 173.4 nm in the case of LNPs 3. All the formulations were characterized by a narrow size distribution (polydispersity index [PI] < 0.2) and a negative ζ (from about −11.6 to −29.4 mV). The use of different lipid molar ratio did not significantly affect the particle size; on the other hand, the higher percentage of the ionizable lipid, namely DODAP, led to an increase of the zeta potential (Table 2). In all cases, the encapsulation efficiency (EE%) of anti-miR103/107 into the LNPs was always very high, especially when a higher percentage of DODAP was used, e.g., LNPs 2 and LNPs 4.

LNPs did not exhibit toxicity in primary rat cortical neurons

In vitro experiments were performed in primary rat cortical neurons to demonstrate the absence of toxicity. To this aim, rat cortical neurons were exposed to different LNP preparations with an initial concentration of 0.7 mg/mL lipids and 0.4 mg/mL anti-miRNA in basal conditions, after 1:1,000, 1:100, and 1:10 v/v dilution, and their effects on mitochondrial redox activity were observed after 24 h of treatment. The results reported in Figure S1 demonstrated the absence of toxicity of empty LNPs 1–4, since no changes in mitochondrial oxidative capacity were observed in basal conditions.

LNPs 1, 3, and 4 encapsulating anti-miRNA103/107 counteracted the impairment of mitochondrial redox activity in cortical neurons exposed to OGD/reoxygenation

In order to demonstrate the effectiveness of LNPs to exert neuroprotection, primary cortical neurons were exposed to OGD followed by reoxygenation (OGD/REOXY) in presence or in absence of LNPs 1–4. The results obtained demonstrated that LNPs treatment was able to prevent the impairment in mitochondrial redox ability occurring in cortical neurons during OGD/REOXY, and that this effect was more evident for cortical neurons exposed to OGD/REOXY in the presence of LNPs 1, 3, and 4 (Figure 1), whereas LNP 2 did not show any effect.

To clarify whether these effects might be related to the ability of LNPs to interfere with NCX 1 expression and activity through the release of anti-miRNA103/107 into neurons, further experiments were performed by using cortical neurons exposed for 6 h to LNPs 1–4 empty or pre-loaded with anti-miRNA103/107. As reported in Figure 2A, the expression of neuronal anti-miRNA did not change following empty LNPs exposure. By contrast, after treatment with anti-mirRNA-103/107-loaded LNPs, the expression of neuronal anti-miRNA significantly and robustly increased (Figure 2B).

Table 2. Characterization of LNP-anti-miR103/107: size, polydispersity index (PI), zeta potential (ζ) encapsulation efficiency (EE%), and anti-miR103/107 actual loading

Formulation	Diameter (nm) ± SD	PI ± SD	ζ (mV ± SD)	Anti-miR103/107 actual loading (nmol/mg lipids ±SD)	EE (% ± SD)
LNP	147.7 ± 11.0	0.2 ± 0.01	−16.8 ± 3.7	–	–
LNP 1	154.3 ± 4.3	0.2 ± 0.03	−29.4 ± 3.5	20.5 ± 0.7	75 ± 2.3
LNP 2	147.4 ± 4.5	0.2 ± 0.03	−19.3 ± 2.6	34.7 ± 0.0	84 ± 2.5
LNP 3	173.4 ± 3.2	0.1 ± 0.04	−26.8 ± 2.4	22.3 ± 0.3	81 ± 3.1
LNP 4	153.3 ± 2.5	0.1 ± 0.02	−11.6 ± 3.2	38.1 ± 0.1	92 ± 2.8

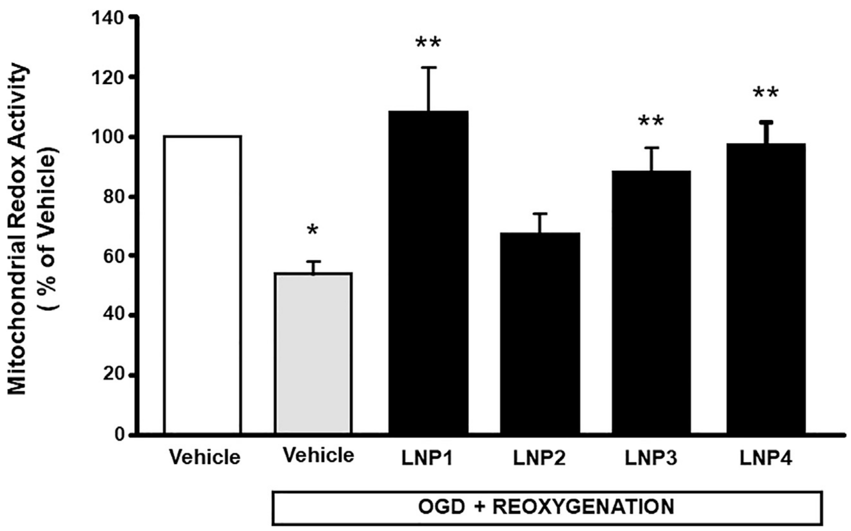

Figure 1. Effect of anti-miRNA103/107-loaded LNP formulations on mitochondrial redox activity in cortical neurons exposed to OGD/reoxygenation

The values for each column represent the mean percentage ± SEM. *p < 0.05 vs. control neurons; **vs. control untreated OGD/REOXY exposed neurons.

Anti-miR-103/107-loaded LNP 3 and 4 treatment significantly reduced ischemic volume in rats subjected to tMCAO

The effect of the four formulations of LNPs containing anti-miRNA103/107 (70 μg/mL of lipids) was assessed on the progression of ischemic damage. Several experimental approaches have been evaluated in terms of duration of treatment and frequency of administrations and the most significant results were obtained when the animals underwent four repeated intravenous (i.v.) infusions: 18 and 24 h before stroke induction, and 1 h and 5 h after ischemia. As shown in Figure 3, the use of LNP 3 and LNP 4 and, to a lesser extent, LNP 1, encapsulating anti-miRNA 103/107 was able to induce a significant reduction of ischemic volume. In order to verify whether LNPs may per se have a neuroprotective role and reduce the ischemic volume, experiments were also carried out in animals receiving LNPs loaded with miRNA scramble. In this experimental subgroup, LNPs were used without dilution from the starting stock. The data obtained did not show any difference in terms of changes in ischemic volume when the effect of LNPs loaded with miRNA scramble at dose 1/1 (n = 4) was compared with the ischemic volume calculated in animals receiving vehicle (n = 3) (Figure S2).

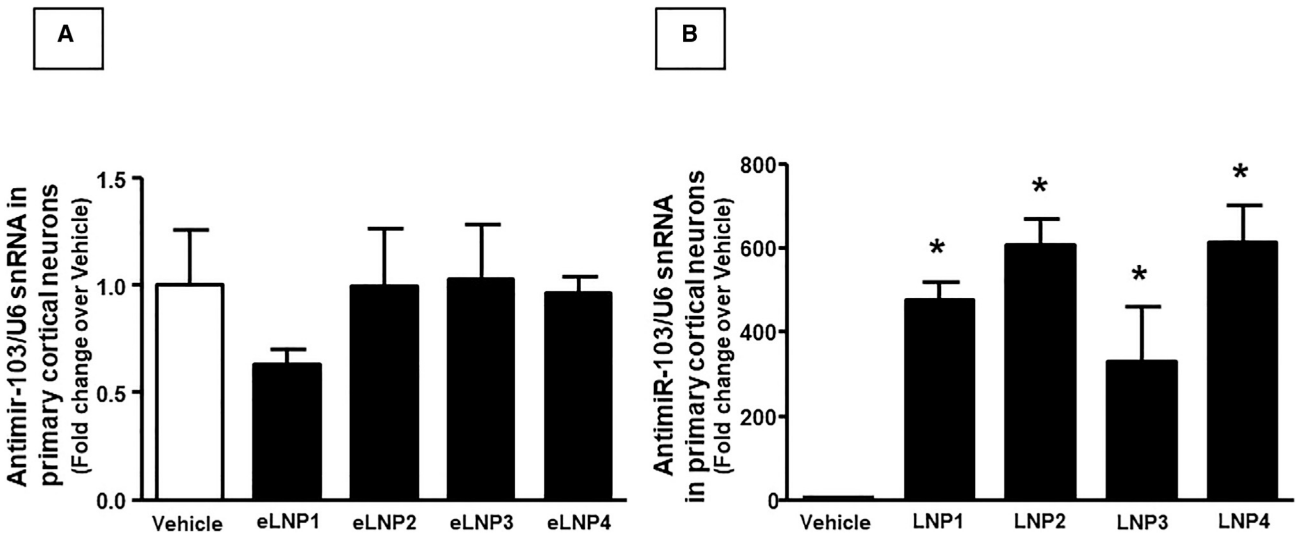

Figure 2. Expression analysis by real-time PCR of anti-miRNA103/107 levels on cortical neurons treated with empty (eLNP) and anti-miR-103/107-loaded LNPs

Anti-microRNA levels are expressed as fold change of relative expression levels over the control group, represented by untreated cortical neurons. Each column represents the mean ± SEM. Results of anti-microRNA expression were normalized with respect to U6 snRNA as internal control. n = 4–5 samples per each group. *p < 0.05 vs. control group.

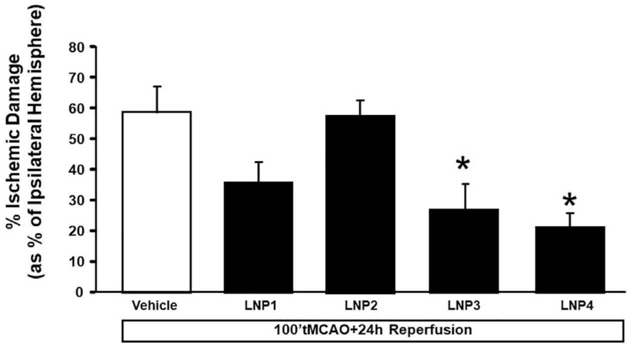

Figure 3. *In vivo* effect of anti-miRNA103/107-loaded LNPs on ischemic volume of rats subjected to tMCAO followed by 24 h from reperfusion
Ischemic damage was assessed in rats subjected to tMCAO and treated with four bolus injections of LNPs through the caudal vein 24 and 18 h before tMCAO induction, and 1 and 5 h after reperfusion. Vehicle group represents animals treated with tMCAO and saline solution. Each column represents the mean ± SEM. $^{*}p < 0.05$ vs. vehicle group.

Treatment with LNP 3 and 4 encapsulating anti-miRNA103/107 prevented NCX1 downregulation in rat cerebral cortex after tMCAO

First, we observed an increase in anti-miRNA103/107 levels in temporoparietal cortex of rats treated with LNP1–4 loaded with anti-miRNA103/107 (Figure 4A). Then, we assessed NCX1 levels in terms of mRNA and protein content to evaluate whether treatment with LNPs might abolish the NCX1 downregulation mediated by cerebral miR-103/107 rise during ischemia in temporoparietal brain cortex of ischemic rats.Indeed, following treatment of ischemic rats with LNPs 1, 3, and 4 encapsulating anti-miRNA103/107, the decrease of ncx1 mRNA induced by tMCAO was prevented (Figure 4C). Similarly, western blot analysis showed that downregulation of NCX1 protein induced by tMCAo and observed in temporoparietal brain cortex of ischemic rats was significantly prevented after LNP 1, 3, and 4 treatments (Figure 4B).

Anti-miRNA-103/107 plasma levels changed over time after i.v. administration of LNP-anti-miRNA 103/107 in non-ischemic animals

In order to verify the permanence in circulation of the nanoencapsulated anti-miRNA administered intravenously, plasma samples were collected from untreated animals (control group) and from animals receiving LNP anti-miRNA as previously described. Real-time PCR analysis showed that anti-miRNA103/107 levels were considerably high up to 96 h after the last administration. At a longer time interval of 7 days, anti-miRNA plasma levels returned to basal (Figure 5A).

Furthermore, to verify that LNP3 loaded with anti-miRNA103/107 reached the brain, anti-miR 103a was labeled with rhodamine in 5′ and encapsulated in nanoparticles.

The rhodamine fluorescence intensity has been quantified in animals treated with 5′-Rho-Anti-miR103a-3′ loaded LNP3 in several organs, as shown in Figure 5B. Interestingly, the fluorescence intensity was higher in brain than in heart, liver, and muscle, thus supporting the hypothesis that transferrin-conjugated LNPs were preferentially localized in the brain.

DISCUSSION

The results of the present study demonstrated for the first time that the encapsulation of anti-miRNA103/107 in transferrin-conjugated stabilized LNPs allowed the BBB crossing and significantly reduced brain ischemic damage.

In particular, here we gave the basis for the development of a new therapeutic strategy for the treatment of cerebral ischemia based on the use of anti-miRNA103/107 incorporated in lipid nanocarriers, which can be intravenously administered. The main element of originality consisted in the proposal of a drug formulation with an innovative pharmacodynamic mechanism to treat a disease for which, at present, there are no other strategies, except for the fibrinolytic agent rTPA or intra-arterial thrombectomy. It should also be stressed that the use of a lipid-based nanocarrier, already used in the treatment of hereditary transthyretin-mediated amyloidosis or for vaccination against COVID-19, is proposed here for the delivery of anti-miRNA into the CNS, representing a strategy capable of generating technological improvements not only in the ischemic cerebral pathology under study, but also in numerous other unexplored neurodegenerative diseases. In fact, the use of anti-miRNAs to treat diseases, such as Parkinson's, Alzheimer's, and amyotrophic lateral sclerosis, is emerging in recent years as a frontier therapy.[13–16] Therefore, an i.v.-administrable formulation able to deliver therapeutic RNA across the BBB to reach the brain regions involved in the pathological process represents a significant advancement toward the research of novel therapeutic strategies.

It should be underlined that drug delivery systems (DDSs) for BBB crossing in stroke and other neurological disorders is a subject of intense and diverse studies.[17] Indeed, numerous papers reported specific, safe, and effective targeted delivery of the DDSs carrying diverse therapeutic agents to the cerebrovascular targets after intravascular injection in animals.[17] An intense and promising field of research is represented by the use of nanoparticles modified with antibodies targeting proteins expressed on endothelium. In this regard, it has been reported that modified urokinase fused with the ligands (antibodies and scFv) binding to the endothelial cell surface determinant PECAM-1 accumulates in the brain, lyses thrombi, and alleviates thrombotic stroke in a mouse model.[18] Another important outcome has been given with DDSs using ligands binding to VCAM-1, which being relatively more selective, appears on the surface of pathologically altered BBB endothelium, thus showing remarkable uptake in the animal brain injury models allowing magnetic resonance imaging of the pathology.[19] More recently, it has been reported that targeting CAMs, especially VCAM, with mAbs and nanocarriers represent a promising direction for innovative stroke therapies.[20]

In our study we propose a new approach favoring BBB crossing and brain accumulation by using transferrin on the LNP surface. We

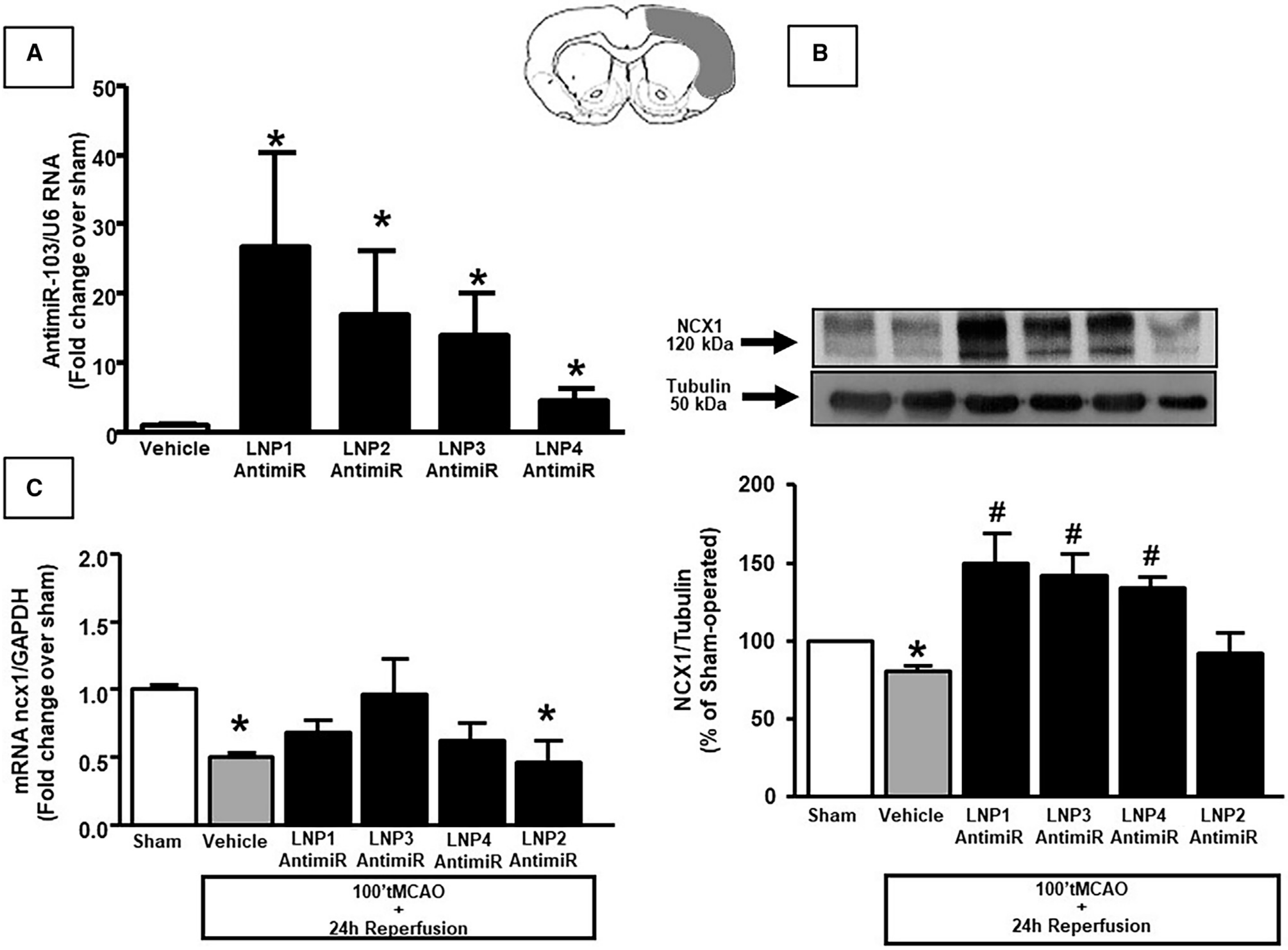

Figure 4. Evaluation of NCX1 expression in samples of ipsilateral cortex from ischemic rats treated with anti-miRNA103/107-loaded LNPs

(A) Expression analysis by real-time PCR of anti-miRNA103/107 levels in temporoparitel cortex of rats treated with anti-miR-103/107-loaded LNP1-4 24 h after i.v. injection. Results were normalized with respect to U6 snRNA as internal control. n = 3 samples per each group. (B) NCX1 protein levels are expressed as percentage vs. the sham-operated controls. Each column represents the mean ± SEM. Results of NCX1 expression were normalized with respect to α-tubulin. On the top, representative blots of NCX1 and α-tubulin signals in the sham and ischemic animals euthanized at 24 h from reperfusion. n = 4 samples per each group. *p < 0.05 vs. sham-operated control group. $^{\#}p < 0.05$ vs. vehicle group. (C) ncx1 mRNA levels are expressed as fold change of relative expression levels over the sham-operated group. Each column represents the mean ± SEM. Results of ncx1 expression were normalized with respect to GAPDH as internal control. n = 3 samples per each group. *p < 0.05 vs. control group.

showed that LNPs represent an additional promising approach to design new therapies based on miRNA in the treatment of ischemia. LNPs are the most investigated platform for RNA delivery with many formulations in clinical trials and three products approved by the Food and Drug Administration,[21] thus offering guarantees for the future scale-up and large-scale production of the formulation in GMP grade. Here, LNPs with different compositions, namely different content of ionizable lipids and conjugation with transferrin, have been tested. All the formulations, used at three different concentrations, showed no toxicity on cortical neurons. Neuroprotection in primary cortical neurons exposed to OGD/REOXY was also evident by using LNP encapsulating anti-miRNA103/107, but only in the case of LNPs 1, 3, and 4. Actually, among all the LNP formulations tested in the present study, LNP 2 is characterized by a higher content of ionizable lipid compared with LNP 1; thus, while the ionizable lipid is the key component to have RNA uptake into cells as well as lysosomal escape, its concentration must be finely tuned up for optimal transfection efficiency. Interestingly, in the case of transferrin-targeted LNPs, both formulations, LNP 3 and 4, provided high levels of neuroprotection, suggesting that the presence of transferrin on the surface should change the mechanism of cell uptake, leading to high neuroprotection independently on the ionizable lipid content. *In vivo* studies demonstrated that animals receiving LNP encapsulating anti-miRNA103/107 showed an ischemic volume significantly lower than untreated animals. Interestingly, the highest reduction in ischemic volume obtained with LNP 3 and 4 could be ascribed to the presence of transferrin on the LNP surface that could facilitate BBB crossing. This peculiar feature may explain why LNP 2 loaded with

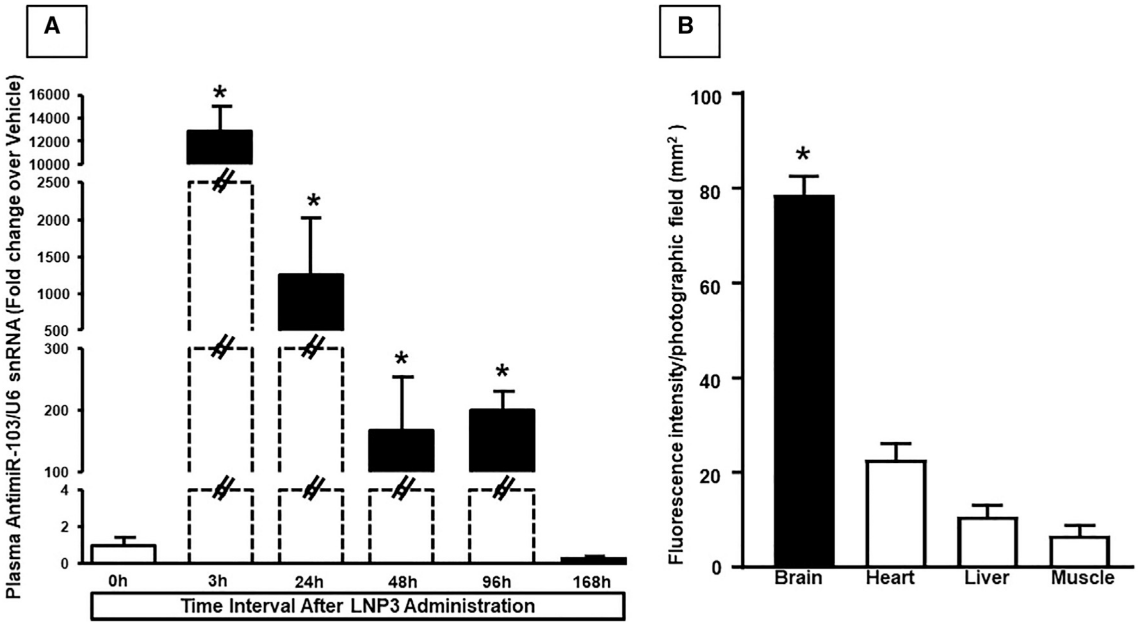

Figure 5. Plasma and organ distribution of aanti-miR-103/107-loaded LNP 3 after i.v. injection

(A) Expression analysis by real-time PCR of circulating anti-miRNA103/107 levels in blood of rats exposed to tMCAO and treated with anti-miR-103/107-loaded LNP 3 at different time intervals from reperfusion. In (B) are reported RHOD-1/Fluorescein fluorescent levels in different organs after i.v. injection of fluorescent LNP3. (A) Anti-microRNA levels are expressed as fold change of relative expression levels over the vehicle group, represented by ischemic rats treated with saline solution. (B) Quantification of fluorescence intensity on tissue sections at the level of the brain cortex, liver, heart and gastrocnemius muscle is expressed in terms of pixel intensity value. Pixels expressing rhodamine co-localizing with fluorescein were identified. Results are expressed in arbitrary units. Each column represents the mean ± SEM. Results of anti-microRNA expression were normalized with respect to U6 snRNA as internal control. n = 3–4 samples per each group. *$p < 0.05$ vs. other experimental groups.

anti-miRNA103/107, which lacks transferrin on their surface, was ineffective *in vivo*, although effective *in vitro*. However, the reduced ischemic volume found at a lesser extent also in the case of transferrin untargeted LNP1 also suggests a partial alteration of BBB in this experimental animal model.

Finally, it should be underlined that plasma levels of anti-miR-103/107 drops 96 h after LNP injection, thus rendering higher the translational potential of this therapeutic approach. This is not surprising because LNPs are designed to be long circulating nanoparticles due to the presence of polyethylenglycol on the nanoparticle surface. In particular, PEGylation of nanoparticles avoids their opsonization leading to a longer blood circulation and to a wide distribution in tissues. Notably, all LNP preparations did not show cytotoxic effects and, more importantly, the neuronal expression of anti-miRNA and miR-103/107 itself did not undergo changes following the exposure of neurons to the four different LNP empty preparations. By contrast, a significant reduction in miR-103/107 levels was observed in neurons exposed to anti-miRNA LNPs, thus confirming the occurrence of the internalization process. Notably, following the systemic administration of LNP 1–4 to ischemic rats, a quantitative increase of anti-miRNA103/107 was observed, the latter was associated with a reduction of miRNA-103/107 in the temporoparietal cortex of rats. As expected, the administration of LNPs containing anti-miRNA103/107 was able to prevent the reduction of the sodium calcium exchanger, NCX1, thus promoting neuronal survival in ischemic conditions.[12]

While the study demonstrated that LNPs encapsulating anti-miRNA103/107 could represent a novel and powerful weapon against ischemic damage, further studies should allow for setting up the optimal therapeutic posology to design a future therapeutic strategy. Another limitation of our approach is represented by the lack of specificity for the ischemic region, as it occurs with DDSs equipped with antibodies against penumbra-related selective targets.[20]

In summary, the present study confirms the important role of the miRNA-103/107 family as a therapeutic target in stroke and validates a potentially exploitable pharmacological strategy not only for the administration of drugs that reach the CNS and are therefore useful for the treatment of ischemic or neurodegenerative pathology, but for any other pathology in which it becomes extremely important to direct the drug at the target organ level, opening new perspectives of pharmacological innovation in different sectors of medical pathology.

MATERIALS AND METHODS

Reagents

Anti-microRNA103/107 (5′-rUrCrArUrArGrCrCrCrUrGrUrArCrAr ArUrGrCrUrGrCrU-3′) and (5′-/5RhoR-XN/rUrCrArUrArGrCrCr CrUrGrUrArCrArArUrGrCrUrGrCrU-3′), namely anti-miR103/107,

was synthesized by Tema Ricerca s.r.l. (Bologna, Italy). 1,2-dioleyl-3-dimethylammonium propane (DODAP), N-palmitoyl-sphingosine-1-succinyl[methoxy(polyethylene glycol)2000] (PEG2000-Cer16) and 1,2-distearoyl-sn-glycero-3-phosphoethanolamine-N-[amino(polyethylene glycol)-2000]-Maleimide (DSPE-PEG-Mal) and 1,2-dioleoyl-sn-glycero-3-phosphoethanolamine-N-(carboxyfluorescein) (ammonium salt) were obtained by Avanti Polar Lipids. Disteroylphosphatidylcholine (DSPC) was kindly offered from Lipoid GmbH (Cam, Switzerland). Cholesterol (CHOL), human transferrin (Tf), ammonium ferrithiocyanate, sodium borate, sodium chloride, sodium citrate, sodium phosphate, HEPES, ethylenediaminetetraacetic acid (EDTA), citric acid, Sepharose G-25, and 2-iminothiolane (Traut's reagent) were purchased from Sigma-Aldrich (USA). Ethanol and other solvents were obtained by Exacta Optech (Italy).

Preparation of LNPs encapsulating anti-miRNA103/107

Lipid nanoparticles encapsulating anti-miRNA103/107 (LNP-anti-miRNA103/107) were prepared as previously described,[22] with some modifications. Briefly, an ethanol stock solution of lipids (40% v/v of total preparation) composed of DSPC/CHOL/DODAP/PEG2000-Cer16 (see Table 1) and an anti-miRNA103/107 citric acid solution (20 mM, pH 4.0), were prepared. To obtain double-marketed LNP, a citric acid solution (20 mM pH 4.0) of anti-miR103/107 sequence labeled with rhodamine dye was used. In addition, the fluorescent lipid 1,2-dioleoyl-sn-glycero-3-phosphoethanolamine-N-(carboxyfluorescein) was added to lipid ethanol solution (0.1% w/w of total lipids).

In the case of LNP-anti-miRNA103/107 decorated with Tf, a lipid composition of DSPC/CHOL/DODAP/PEG2000-Cer16/DSPE-PEG 2000-Mal (see Table 1) was used. Then, the lipid ethanol solution was added to the buffer solution under stirring; the resulting suspension was dimensioned by a thermos barrel extruder system (Northern Lipids Inc., Vancouver, BC, Canada), maintained at 65°C, repeatedly passing the suspension through polycarbonate membranes with decreasing pore sizes (Nucleopore Track Membrane 25 mm, Whatman, Brentford, UK). The preparation was then dialyzed (3.5 kDa cutoff) against 20 mM citrate buffer (pH 4.0 for 1 h) to remove the excess of ethanol and against HBS (20 mM HEPES, 145 mM NaCl, pH 7.4 for 12–18 h), to remove the citrate buffer and to neutralize the LNP surface charge. For Tf-LNP, Tf was first thiolated using 2-iminothiolane (Traut's reagent). Briefly, Tf was dissolved in 0.1M Na-borate buffer pH 8, followed by the addition of Traut's reagent (1:40 mol/mol). After 60-min incubation at room temperature, the excess Tf was removed by molecular exclusion chromatography, Sepharose G-25 column. Thereafter, thiolate-Tf was incubated with preformed LNPs-miR-103/107 (DSPC/CHOL/DODAP/PEG2000-Cer16/DSPE-PEG2000-Mal) overnight at 25°C. The unconjugated Tf and non-encapsulated miRNA were removed by ultracentrifugation at 80,000 rpm at 4°C for 40 min (Optima Max E, Beckman Coulter, USA; rotor TLA 120.2). Blank LNPs were also prepared and used as control. All LNP formulations were prepared in triplicate.

LNP characterization

Size, PI, and superficial charge of LNPs

The mean diameter, PI, and zeta potential (ζ) of LNPs were determined by Zetasizer Ultra (Malvern Panalytical, United Kingdom). Results were averaged over three measurements from independent batches.

Lipid dosage in LNPs

The amount of phospholipids in the LNP-anti-miR-103/107 was determined by the Stewart assay.[23] Briefly, an aliquot of the LNPs was added to a two-phase system, consisting of an aqueous ammonium ferrothiocyanate solution (0.1 N) and chloroform. Each tube was mixed on vortex and then centrifugated for 5 min. The chloroform phase was collected and the concentration of DSPC was obtained by measure of the absorbance at 485 nm with an ultraviolet-visible spectrophotometer (UV VIS 1204; Shimadzu Corporation, Kyoto, Japan). The concentration of the total lipids content was calculated considering a constant ratio between the lipids.

Anti-miR-103/107 encapsulation in LNPs

The amount of anti-miRNA103/107 encapsulated into the LNPs was determined spectrophotometrically. Briefly, an aliquot of the formulation was dissolved in methanol (1:100 v/v) and samples were centrifugated for 30 min at 13,000 rpm (MIKRO 20; Hettich, Tuttlingen, Germany). The supernatants were analyzed by UV (UV-1800, UV Spectrophotometer) at the wavelength of 260 nm. The amount of anti-miRNA loaded into the nanocarriers was expressed as anti-miRNA encapsulation efficiency (EE %), calculated as % ratio between anti-miRNA actual loading (mg of anti-miRNA/mg of total lipids) and anti-miRNA theoretical loading in formulation. For each formulation, results were calculated as the mean of the measures obtained in three different batches (n = 3).

Rat primary cortical neurons

Rat primary cortical neurons were prepared from 17-day-old Wistar rat embryos (Charles River).[24] Briefly, rats were first anesthetized and then decapitated to minimize animal's pain and distress. Dissection and dissociation were performed in Ca^{2+}\Mg^{2+}-free phosphate-buffered saline (PBS) containing glucose (30 mM). Tissues were incubated with papain for 10 min at 37°C and dissociated by trituration in Earle's Balanced Salt Solution containing DNase (0.16 U\mL), bovine serum albumin (10 mg\mL), and ovomucoid (10 mg\mL). Neurons were plated in plastic Petri dishes (Falcon Becton-Dickinson) pre-coated with poly-D-lysine (20 μg\mL) and were grown in MEM\F12 (Life Technologies) containing glucose, 5% of deactivated fetal bovine serum, and 5% of horse serum (Life Technologies), glutamine (2 mM\L), penicillin (50 U\mL), and streptomycin (50 μg\mL) (Invitrogen). Within 48 h of plating, cytosine arabinoside (ara-C) (10 μM) was added to prevent non-neuronal cell growth. Neurons were cultured at 37 °C in a humidified 5% CO_2 atmosphere and used after 7–10 days of culture. Cell density was 5×10^6 cells/well of 60 mm for analysis of qRT-PCR and 15×10^6 cells/well of 100 mm for western blot analysis.

In vivo experimental groups

Ninety Sprague-Dawley male rats (Charles River Laboratories, Calco, Varese, Italy) weighing 200 to 250 g were housed under diurnal

lighting conditions (12-h darkness/light). It has been calculated that about 20% of the animals used were excluded from the experimental groups due to the absence of ischemic lesions (10%) or to mortality related to the experimental procedure (10%). Animals were randomly allocated in the different experimental groups, and the treatment with LNP formulations was blindly performed, since each formulation was labeled with a number by a researcher different from the one performing the treatment. Furthermore, the collected samples were identified with a numeric code and all the postmortem experiments were performed by a researcher blinded to the applied treatment. The key to open the blinding was provided only after the analysis was concluded. Finally, sample size was a priori determined by G-power software. Experiments were performed according to the international guidelines for animal research. The experimental protocol was approved by the Animal Care Committee of the "Federico II" University of Naples.

Transient focal ischemia and evaluation of infarct volume

Transient focal ischemia was induced, as previously described[25]. In brief, occlusion of the middle cerebral artery (MCA) was performed in male rats anesthetized using a mixture of oxygen and sevoflurane at 3.5% (Medical Oxygen Concentrator LFY-I-5A). A 5-O surgical monofilament nylon suture (Doccol, Sharon, MA) was inserted from the external carotid artery into the internal carotid artery and advanced into the circle of Willis up to the branching point of the MCA, thereby occluding the MCA. Achievement of ischemia was confirmed by monitoring regional cerebral blood flow in the area of the right MCA. Cerebral blood flow was monitored through a disposable microtip fiberoptic probe (diameter 0.5 mm) connected through a Master Probe to a laser Doppler computerized main unit (PF5001; Perimed, Järfälla, Sweden) and analyzed using PSW Perisoft 2.5.[26] Animals not showing a cerebral blood flow reduction of at least 70% were excluded from the experimental group, as well as animals that died after ischemia induction. Rectal temperature was maintained at 37 ± 0.5°C with a thermostatically controlled heating pad and lamp. All surgical procedures were performed under an operating stereomicroscope in a blind manner.

Animals were killed with sevoflurane overdose 24 h after ischemia. Brains were quickly removed, sectioned coronally at 1-mm intervals, and stained by immersion in the vital dye (2%) 2,3,5-triphenyltetrazolium hydrochloride. The infarct volume was calculated by summing the infarction areas of all sections and by multiplying the total by slice thickness.[26] To avoid that edema could affect the infarct volume value, infarct volume was expressed as percentage of the ischemic damage by dividing the infarct volume by the total ipsilateral hemispheric volume.

Experimental *in vivo* drug administration

LNP nanovectors, empty, or carrying the selected anti-miRNA, were administered in rats through the tail vein. For each experimental group, i.v. administration of 100 μL of the different LNPs, diluted to the appropriate concentration by adding saline solution (0.9% NaCl, pH 7.4), was carried out as a bolus, using a 1-mL insulin syringe (BD Plastics). Ischemic control animals received only saline administration. The animals received four administrations, 24 and 18 h before ischemia and 1 and 5 h after tMCAO induction.

LNPs loaded with rhodamine-labeled anti-miRNA were injected in non-ischemic animals four times at the same time intervals and in the same amount of non-labeled LNPs administered to ischemic animals.

Anti-miRNA levels were evaluated in the temporoparietal cortex of rats injected with anti-miRNA, 24 h after the last injection. Anti-miRNA levels were also in plasma obtained from blood (500 μL) withdrawn from tail vein at different time intervals after LNP injection: 3, 24, 48, 96, and 168 h. To dilate the blood vessels, rat tails were immersed in preheated water at 38°C for 40–50 s. Blood was collected into purple top EDTA tubes and centrifuged (2,000 rpm) at 4°C for 20 min. After centrifugation, plasma was collected into 1.5-mL Eppendorf tubes labeled with tracking number and "plasma."

Fluorescence intensity quantification

Animals were anesthetized and transcardially perfused with saline solution containing 0.01 mL heparin (10 U/mL heparin in 0.1 M PBS) followed by 60 mL of 4% paraformaldehyde. The brain, liver, and heart were rapidly removed on ice and postfixed overnight at +4°C and cryoprotected in 30% sucrose in 0.1 M phosphate buffer (PB) with sodium azide 0.02% for 24 h at 4°C. The gastrocnemius was frozen with liquid nitrogen and stored at −80°C. The organs were then sectioned frozen on a sliding cryostat at 10-μm thickness. Subsequently, after mounting the slides, they were observed directly under the confocal microscope (Zeiss LSM 700).

Quantification of fluorescence intensity on tissue sections at the level of brain cortex, liver, heart, and gastrocnemius muscle was done in terms of pixel intensity value by using the NIH image software. Briefly, digital images were taken with ×20 objective and identical laser power settings and exposure times were applied to all the photographs from each experimental set. Images were first thresholded to identify the positive signal; subsequently, the pixels expressing rhodamine co-localizing with fluorescein were identified. Results were expressed in arbitrary units. n = 3 mice per group.

Western blot analysis

Samples from cortical neurons and rat ischemic brain regions were homogenized in a lysis buffer (50 mmol/L Tris-HCl, pH 7.5, 100 mmol/L NaCl, 1% Triton X-100) containing protease and the phosphatase inhibitor. After centrifugation at 12,000 × *g* at 4°C for 15 min, the supernatants were collected. Protein concentration was estimated using the Bradford method, by means of a spectrophotometer (Eppendorf). Then, 80–100 μg of protein lysate was mixed with a Laemmli sample buffer and boiled at 95°C for 5 min. The samples were resolved by sodium dodecyl sulfate polyacrylamide gel electrophoresis and transferred to nitrocellulose membranes. Blots were probed with antibodies for NCX1 (Swant, rabbit polyclonal, 1:1,000), and α-tubulin (Abcam, mouse monoclonal, 1:10,000) diluted in tris-buffered saline (TBS-T) 1% bovine serum albumin overnight (4°C). Then, they were detected using horseradish peroxidase conjugated secondary antibody (mouse

and rabbit, Cell Signaling; 60 min at room temperature in 5% non-fat milk) and an enhanced luminescence kit (Amersham Pharmacia Biotech, NJ, USA).

Real-time PCR

Total RNA from neuronal cultures or brain tissues was extracted by using Trizol following the supplier's instructions (TRI Reagent, Sigma). For miRNA analysis, 5 ng of RNA was specifically retrotranscribed in the target cDNA, using High-Capacity cDNA Reverse Transcription Kit (Applied Biosystems) and Taqman probes, following TaqMan Small RNA Assays Protocol (16°C for 30 min, 42°C for 30 min, and 85°C for 5 min). Regarding mRNA analysis, 1 μg of total RNA was retrotranscribed, following the manufacturer's protocol: 25°C for 10 min, 37°C for 120 min, and 85°C for 5 min. Quantitative real-time PCR was performed with TaqMan Universal PCR Master Mix II (Applied Biosystems) in a 7500 Fast Real-Time PCR System (AB Applied Biosystems). cDNA samples were amplified simultaneously in triplicate in one assay run, following the protocol for Taqman assays: 50°C for 2 min, 95°C for 10, 40 cycles of amplification of 95°C for 15 s, and 60°C for 1 min. All reactions were run in triplicate. Results were analyzed and exported with 7500 Fast System SDS Software. Taqman probes used are the following: miRNA Assay anti-miRNA103/107-3p (ID: 121115_mat); miRNA Control Assay U6 snRNA (ID: 001973); Taqman Assay Slc8a1 (Rn01472843_m1); Taqman Assay Gapdh (Rn99999916_s1).

OGD and REOXY

Cortical neurons were first exposed to OGD for 3 h and then to reoxygenation for 24 h.[27,28] In brief, the culture medium was replaced with a hypoxia medium, which was previously saturated with 95% N_2 and 5% CO_2 for 20 min; it contained NaCl 116 mM, KCl 5.4 mM, $MgSO_4$ 0.8 mM, $NaHCO_3$ 26.2 mM, NaH_2PO_4 1 mM, $CaCl_2$ 1.8 mM, glycine 0.01 mM, and 0.001 w/v phenol red. Hypoxic conditions were maintained with a hypoxia chamber (temperature 37°C, atmosphere 95% N_2, and 5% CO_2). These experimental conditions induced a 30% decrease of pO_2 in the medium. Deprivation of oxygen and glucose was stopped by placing the cells in the regular culture medium saturated with a mixture of 95% O_2 and 5% CO_2 for 10 min. Reoxygenation was achieved by returning neurons to normoxic conditions (37°C in a humidified 5% CO_2 atmosphere) for 24 h.

Determination of mitochondrial oxidative activity

Mitochondrial function was assessed by measuring the level of mitochondrial dehydrogenase activity using the reduction of 3-(4,5-dimethylthiazol-2-yl)-2,5, diphenyltetrazolium bromide (MTT) as substrate.[28] The assay is based on the ability of living mitochondria to convert dissolved MTT into insoluble formazan. Briefly, after treatments, the medium was removed, and the cells were incubated in 500 μM of MTT solution (0.5 mg/mL) for 1 h in a humidified 5% CO_2 incubator at 37°C. The incubation was then stopped by removing the medium and by adding 1 mL of DMSO to solubilize the formazan. The absorbance was detected at 540 nm. Data were expressed as the percentage of mitochondrial redox activity compared with untreated cultures.

Statistical analysis

Values were expressed as means ± standard error of the mean (SEM). Real-time PCR results were expressed as fold change ($2^{-\Delta\Delta Ct}$) compared with the control group settled to 1, following the instructions provided by the literature.[24] Briefly, difference between Ct values of gene of interest and internal control (ΔCt) was calculated for both control sample and target sample. Then, difference between ΔCt of target sample and control sample (ΔΔCt) was calculated. Fold change of gene expression of target samples compared with control sample is calculated as $2^{-\Delta\Delta Ct}$. Statistical analysis was performed with GraphPad Prism 5.0 (GraphPad Software, Inc., San Diego, CA), using one-way analysis of variance followed by Newman-Keuls posttest for group more than two. To compare two groups, unpaired t test was used. Statistical significance was accepted at the 95% confidence level ($p < 0.05$).

Statistical analysis for *in vitro* experiments was performed by using one-way analysis of variance followed by Newman-Keuls. The MTT experiments were performed in triplicate and the values expressed as percentage of mean ± SEM.

DATA AND CODE AVAILABILITY

The authors confirm that the data supporting the findings of this study are available within the article [and/or] its supplemental information.

SUPPLEMENTAL INFORMATION

Supplemental information can be found online at https://doi.org/10.1016/j.omtn.2024.102131.

ACKNOWLEDGMENTS

This work was supported by grant CN00000041 "National Center for Gene Therapy and Drugs based on RNA Technology" (concession number 1035 of 17 June 2022-PNRR MUR – M4C2 – Investment 1.4 Call "National Centers", financed by EU- NextGenerationEU), code project (CUP) E63C22000940007 to G.P., G.D.R., and O.C. We also acknowledge PON NEON (ARS01_00769) from the Italian Ministry of Research, MIUR, to G.P.; NanomiRNICTUS Project from Ministry of Economic Development (MISE) to L.A.

AUTHOR CONTRIBUTIONS

Conceptualization: L.A., A.S., G.D.R., G.P.; Methodology: O.C., P.B., P.C., A.V., A.C., V.C., V.N., M.J.S.; Formal analysis and investigation: O.C., P.B., P.C., A.V., V.C., V.N., M.J.S., S.A., and V.V.; Writing - original draft preparation: P.C., G.P., L.A., G.D.R., A.S.; Funding acquisition: G.P., L.A., G.D.R., and O.C.

DECLARATION OF INTERESTS

The authors declare no competing interests.

REFERENCES

1. (2000). Effect of intravenous recombinant tissue plasminogen activator on ischemic stroke lesion size measured by computed tomography. NINDS; The National Institute of Neurological Disorders and Stroke (NINDS) rt-PA Stroke Study Group. Stroke *31*, 2912–2919.

2. Saugstad, J.A. (2010). MicroRNAs as effectors of brain function with roles in ischemia and injury, neuroprotection, and neurodegeneration. J. Cerebr. Blood Flow Metabol. *30*, 1564–1576.

3. Sun, P., Liu, D.Z., Jickling, G.C., Sharp, F.R., and Yin, K.J. (2018). MicroRNA-based therapeutics in central nervous system injuries. J. Cerebr. Blood Flow Metabol. *38*, 1125–1148.

4. Hennig, T., Prusty, A.B., Kaufer, B.B., Whisnant, A.W., Lodha, M., Enders, A., Thomas, J., Kasimir, F., Grothey, A., Klein, T., et al. (2022). Selective inhibition of miRNA processing by a herpesvirus-encoded miRNA. Nature *605*, 539–544.

5. Selbach, M., Schwanhäusser, B., Thierfelder, N., Fang, Z., Khanin, R., and Rajewsky, N. (2008). Widespread changes in protein synthesis induced by microRNAs. Nature *455*, 58–63.

6. Pignataro, G., Sirabella, R., Anzilotti, S., Di Renzo, G., and Annunziato, L. (2014). Does Na(+)/Ca(2)(+) exchanger, NCX, represent a new druggable target in stroke intervention? Transl. Stroke Res. *5*, 145–155.

7. Cuomo, O., Vinciguerra, A., Cerullo, P., Anzilotti, S., Brancaccio, P., Bilo, L., Scorziello, A., Molinaro, P., Di Renzo, G., and Pignataro, G. (2015). Ionic homeostasis in brain conditioning. Front. Neurosci. *9*, 277.

8. Magli, E., Fattorusso, C., Persico, M., Corvino, A., Esposito, G., Fiorino, F., Luciano, P., Perissutti, E., Santagada, V., Severino, B., et al. (2021). New Insights into the Structure-Activity Relationship and Neuroprotective Profile of Benzodiazepinone Derivatives of Neurounina-1 as Modulators of the Na(+)/Ca(2+) Exchanger Isoforms. J. Med. Chem. *64*, 17901–17919.

9. Severino, B., Corvino, A., Fiorino, F., Frecentese, F., Perissutti, E., Caliendo, G., Santagada, V., Magli, E., Molinaro, P., Pignataro, G., et al. (2019). Development, Validation of LC-MS/MS Method and Determination of Pharmacokinetic Parameters of the Stroke Neuroprotectant Neurounina-1 in Beagle Dog Plasma After Intravenous Administration. Front. Pharmacol. *10*, 432.

10. Molinaro, P., Cantile, M., Cuomo, O., Secondo, A., Pannaccione, A., Ambrosino, P., Pignataro, G., Fiorino, F., Severino, B., Gatta, E., et al. (2013). Neurounina-1, a novel compound that increases Na+/Ca2+ exchanger activity, effectively protects against stroke damage. Mol. Pharmacol. *83*, 142–156.

11. Brancaccio, P., Anzilotti, S., Cuomo, O., Vinciguerra, A., Campanile, M., Herchuelz, A., Amoroso, S., Annunziato, L., and Pignataro, G. (2022). Preconditioning in hypoxic-ischemic neonate mice triggers Na(+)-Ca(2+) exchanger-dependent neurogenesis. Cell Death Dis. *8*, 318.

12. Vinciguerra, A., Formisano, L., Cerullo, P., Guida, N., Cuomo, O., Esposito, A., Di Renzo, G., Annunziato, L., and Pignataro, G. (2014). MicroRNA-103-1 selectively downregulates brain NCX1 and its inhibition by anti-miRNA ameliorates stroke damage and neurological deficits. Mol. Ther. *22*, 1829–1838.

13. Broderick, J.A., and Zamore, P.D. (2011). MicroRNA therapeutics. Gene Ther. *18*, 1104–1110.

14. Wang, Z. (2011). The concept of multiple-target anti-miRNA antisense oligonucleotide technology. Methods Mol. Biol. *676*, 51–57.

15. Wu, W. (2010). MicroRNA: potential targets for the development of novel drugs? Drugs R *10*, 1–8.

16. Weiler, J., Hunziker, J., and Hall, J. (2006). Anti-miRNA oligonucleotides (AMOs): ammunition to target miRNAs implicated in human disease? Gene Ther. *13*, 496–502.

17. Teleanu, R.I., Preda, M.D., Niculescu, A.G., Vladâcenco, O., Radu, C.I., Grumezescu, A.M., and Teleanu, D.M. (2022). Current Strategies to Enhance Delivery of Drugs across the Blood-Brain Barrier. Pharmaceutics *14*, 987.

18. Danielyan, K., Ding, B.S., Gottstein, C., Cines, D.B., and Muzykantov, V.R. (2007). Delivery of anti-platelet-endothelial cell adhesion molecule single-chain variable fragment-urokinase fusion protein to the cerebral vasculature lyses arterial clots and attenuates postischemic brain edema. J. Pharmacol. Exp. Therapeut. *321*, 947–952.

19. Gauberti, M., Montagne, A., Marcos-Contreras, O.A., Le Béhot, A., Maubert, E., and Vivien, D. (2013). Ultra-sensitive molecular MRI of vascular cell adhesion molecule-1 reveals a dynamic inflammatory penumbra after strokes. Stroke *44*, 1988–1996.

20. Nong, J., Glassman, P.M., Reyes-Esteves, S., Descamps, H.C., Shuvaev, V.V., Kiseleva, R.Y., Papp, T.E., Alameh, M.G., Tam, Y.K., Mui, B.L., et al. (2023). Targeting lipid nanoparticles to the blood brain barrier to ameliorate acute ischemic stroke. Preprint at bioRxiv. https://doi.org/10.1101/2023.06.12.544645.

21. Yan, Y., Liu, X.Y., Lu, A., Wang, X.Y., Jiang, L.X., and Wang, J.C. (2022). Non-viral vectors for RNA delivery. J. Contr. Release *342*, 241–279.

22. Campani, V., Salzano, G., Lusa, S., and De Rosa, G. (2016). Lipid Nanovectors to Deliver RNA Oligonucleotides in Cancer. Nanomaterials *6*, 131.

23. Stewart, J.C. (1980). Colorimetric determination of phospholipids with ammonium ferrothiocyanate. Anal. Biochem. *104*, 10–14.

24. Sisalli, M.J., Secondo, A., Esposito, A., Valsecchi, V., Savoia, C., Di Renzo, G.F., Annunziato, L., Scorziello, A., and Scorziello, A. (2014). Endoplasmic reticulum refilling and mitochondrial calcium extrusion promoted in neurons by NCX1 and NCX3 in ischemic preconditioning are determinant for neuroprotection. Cell Death Differ. *21*, 1142–1149.

25. Pignataro, G., Studer, F.E., Wilz, A., Simon, R.P., and Boison, D. (2007). Neuroprotection in ischemic mouse brain induced by stem cell-derived brain implants. J. Cerebr. Blood Flow Metabol. *27*, 919–927.

26. Vinciguerra, A., Cepparulo, P., Anzilotti, S., Cuomo, O., Valsecchi, V., Amoroso, S., Annunziato, L., and Pignataro, G. (2020). Remote postconditioning ameliorates stroke damage by preventing let-7a and miR-143 up-regulation. Theranostics *10*, 12174–12188.

27. Goldberg, M.P., and Choi, D.W. (1993). Combined oxygen and glucose deprivation in cortical cell culture: calcium-dependent and calcium-independent mechanisms of neuronal injury. J. Neurosci. *13*, 3510–3524.

28. Sisalli, M.J., Della Notte, S., Secondo, A., Ventra, C., Annunziato, L., and Scorziello, A. (2022). L-Ornithine L-Aspartate Restores Mitochondrial Function and Modulates Intracellular Calcium Homeostasis in Parkinson's Disease Models. Cells *11*, 2909.

OMTN, Volume *35*

Supplemental information

Anti-miRNA103/107 encapsulated in transferrin-conjugated lipid nanoparticles crosses blood-brain barrier and reduces brain ischemic damage

Pasquale Cepparulo, Ornella Cuomo, Virginia Campani, Antonio Vinciguerra, Maria Josè Sisalli, Valeria Nele, Serenella Anzilotti, Valeria Valsecchi, Antonella Casamassa, Paola Brancaccio, Antonella Scorziello, Giuseppe De Rosa, Lucio Annunziato, and Giuseppe Pignataro

Figure S1

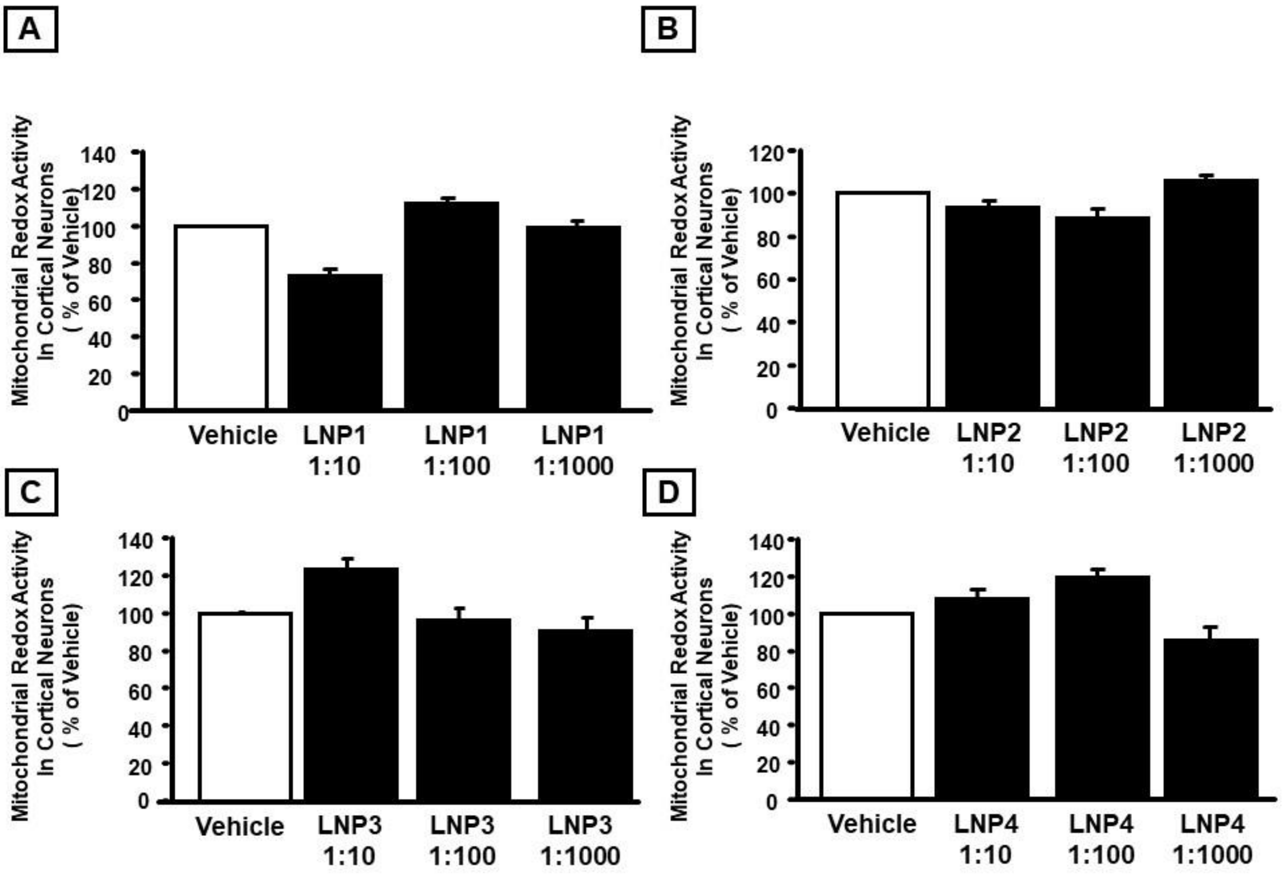

Figure S1. Effect of the different LNPs formulations at 1/100, 1/100 and 1/10 dilution filled with miRNA scramble on Mitochondrial Redox Activity in Cortical Neurons.

Figure S2

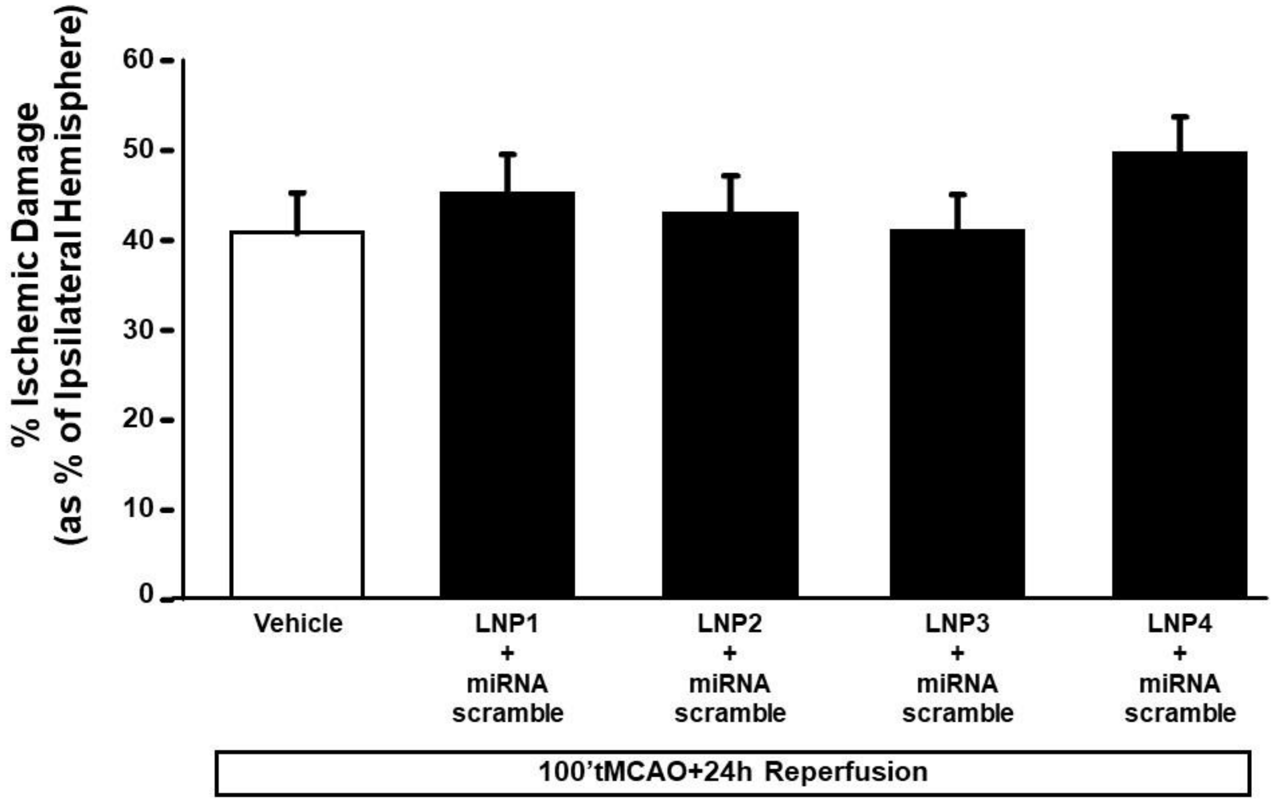

Figure S2. In vivo effect of LNP1-4 filled with miRNA scramble on ischemic volume of rats subjected to tMCAO followed by 24 hours from reperfusion.

Ischemic damage was assessed in rats subjected to tMCAO and treated with four in bolus injections of LNPs through the caudal vein 24 and 18 hours before tMCAO induction, and 1 and 5 hours after reperfusion. Vehicle group represents animals treated with tMCAO and saline solution. Each column represents the mean ± S.E.M.

Figure S3

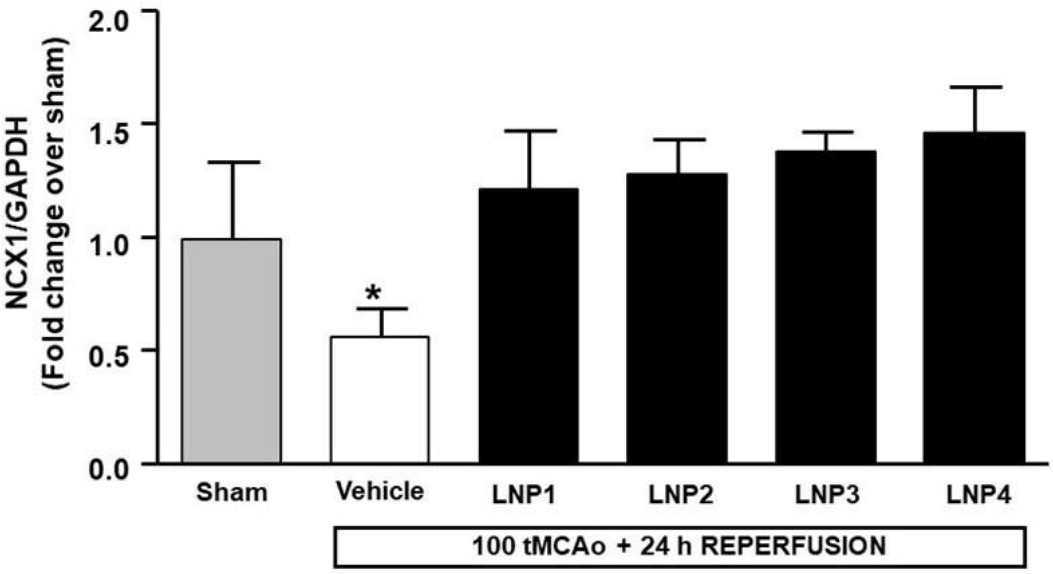

Figure S3. Evaluation of NCX1 expression in samples of ipsilateral striatum from ischemic rats treated with anti-miRNA103/107-loaded LNPs.

ncx1 mRNA levels are expressed as fold change of relative expression levels over the sham-operated group. Each column represents the mean ± S.E.M. Results of ncx1 expression were normalized with respect to GAPDH as internal control. n=3 samples per each group.

*: $p<0.05$ Vs control group.

Table S1: Original data corresponding to data presented in Figures 2, 4 and 5

Fig.2A

Vehicle	0.9±0.2
LNP1	0.57±0.06
LNP2	0.91±0.2
LNP3	0.93±0.23
LNP4	0.87±0.071

Fig.2B

Vehicle	4.4±1.2
LNP1	2128±193
LNP2	2702±279
LNP3	1472±582
LNP4	2732±394

Fig.4A antimiR expression in cortex

Vehicle	0.77±0.2
LNP1	20.9±10.5
LNP2	13.2±7.2
LNP3	10.9±4.7
LNP4	2.6±1.1

Fig.4C NCX1 mRNA expression in cortex

Sham	1.04±0.03
Vehicle+tMCAO	0.47±0.01
LNP1+tMCAO	0.71±0.09
LNP2+tMCAO	0.48±0.16
LNP3+tMCAO	1.01±0.27
LNP4+tMCAO	0.48±0.16

Fig5A AntimiR expression in plasma

0	1.93±0.9
3h	24875±4241
24h	2420±11.91
48h	322±168
96h	388±58
168h	0.6±0.24
